# Supplementary material for: Skin irritation testing using human iPSCs derived 3D skin equivalent model
Source: PLoS One. 2025 Aug 18;20(8):e0330306. doi: 10.1371/journal.pone.0330306 (PMC12360594; doi:10.1371/journal.pone.0330306)
Supplement: S1 File — S1 Table. Infromation of primers. Genes: GAPDH (housekeeping gene for control), puripotency marekrs (OCT4 and SOX2), fibroblast-specific markers (PDGFRα, Col3A1, and Fibronectin), keratinocyte-specific markers (KRT14 and ΔNp63). S1 Fig. IF staining for purity check in hFIBRO and hKERA. (A) Staining for vimentin, a fibroblast-specific marker, in hFIBRO and distribution of vimentin+ cells per fields (n = 3). (B) Staining for ΔNp63, a keratinocyte-specific marker, in hKERA and distribution of ΔNp63 + cells per fields (n = 3). White arrows: DAPI where each marker is not expressed. Scale bar: 100 µm. S2 Fig. Electrophoresis analysis for pluripotency and skin cell-specific markers by hiPSC, hFIBRO, and hKERA. Genes: GAPDH (housekeeping gene for control), puripotency marekrs (OCT4 and SOX2), fibroblast-specific markers (PDGFRα, Col3A1, and Fibronectin), keratinocyte-specific markers (KRT14 and ΔNp63). (DOCX) [file pone.0330306.s001.docx]

**Skin Irritation Testing using Human iPSCs derived 3D Skin Equivalent Model**

Hyewon Shin^1, &^, Se-Eun Kim^1^, C-Yoon Kim^1^, Suemin Lee^1^, Ji-Heon Lee^2^, Jieun Baek^2^, Gujin Chung^2^, Min Woo Kim^1^, Jeong-Seop Oh^3^, Shinhye Park^1^, Yun Hyeong Lee^1^, Youngin Jeong^1^, Jeong Hwan Park^1^, Yoonseo Kim^1^, Myeonghee Lee^1^, Seul-Gi Lee^1, *^, and Hyung Min Chung^4, 5, *^

^1^ College of Veterinary Medicine, Konkuk University, Seoul 05029, Republic of Korea

^2^ R&D Center, CLECELL Inc.,127, Beobwon-ro, Songpa-gu, Seoul, Korea

^3^ Department of Veterinary Pathology, College of Veterinary Medicine, Seoul National University, 1, Gwanak-Ro, Gwanak-Gu, Seoul, 08826, Republic of Korea

^4^ Department of Stem Cell Biology, School of Medicine, Konkuk University, 120 Neungdong-Ro, Gwangjin-Gu, Seoul, Republic of Korea, 05029

^5^ Miracell Bio CO. Ltd., Seoul 04795 Korea

^&^ This author contributed to this work as first author.

^*^ These authors contributed equally to this work as corresponding authors, respectively.

*** Corresponding Author**

: Seul-Gi Lee, PhD

College of Veterinary Medicine, Konkuk University, Seoul, 05029, Republic of Korea.; E-mail: maxwisdom@konkuk.ac.kr

: Hyung Min Chung, PhD

Department of Stem Cell Biology, School of Medicine, Konkuk University, 120 Neungdong-Ro, Gwangjin-Gu, Seoul Republic of Korea, 05029; E-mail: [hmchung@kku.ac.kr](mailto:hmchung@kku.ac.kr)

| Gene name | Direction | Primer sequence (5’ - 3’) | Size (base pair) |
| --- | --- | --- | --- |
| GAPDH | Forward  Reverse | TGGAAATCCCATCACCATCT  TTCACACCCATGACGAACAT | 198 |
| SOX2 | Forward  Reverse | TGGAAACTTTTGTCGGAGA  GCGTGTACTTATCCTTCTTC | 145 |
| OCT4 | Forward  Reverse | AACTCGAGCAATTTGCCAAGCCC  TTCGGGCACTGCAGGAACAAATTC | 328 |
| PDGFR-α | Forward  Reverse | GACTTTCGCCAAAGTGGAGGAG  AGCCACCGTGAGTTCAGAACGC | 121 |
| Col3A1 | Forward  Reverse | CGCCCTCCTAATGGTCAAGG  TTCTGAGGACCAGTAGGGCA | 161 |
| Fibronectin | Forward  Reverse | GCCTTCAAGTTCCCCTGTTAC  GACTCTCTCCGCTTGGATTCT | 176 |
| KRT14 | Forward  Reverse | GCAGTCATCCAGAGATGTGACC  GGGATCTTCCAGTGGGATCT | 181 |
| ΔNP63 | Forward  Reverse | GGAAAACAATGCCCAGACTC  GTGGAATACGTCCAGGTGGC | 294 |

**Supplementary table 1. Infromation of primers.** Genes: GAPDH (housekeeping gene for control), puripotency marekrs (OCT4 and SOX2), fibroblast-specific markers (PDGFRα, Col3A1, and Fibronectin), keratinocyte-specific markers (KRT14 and ΔNp63).


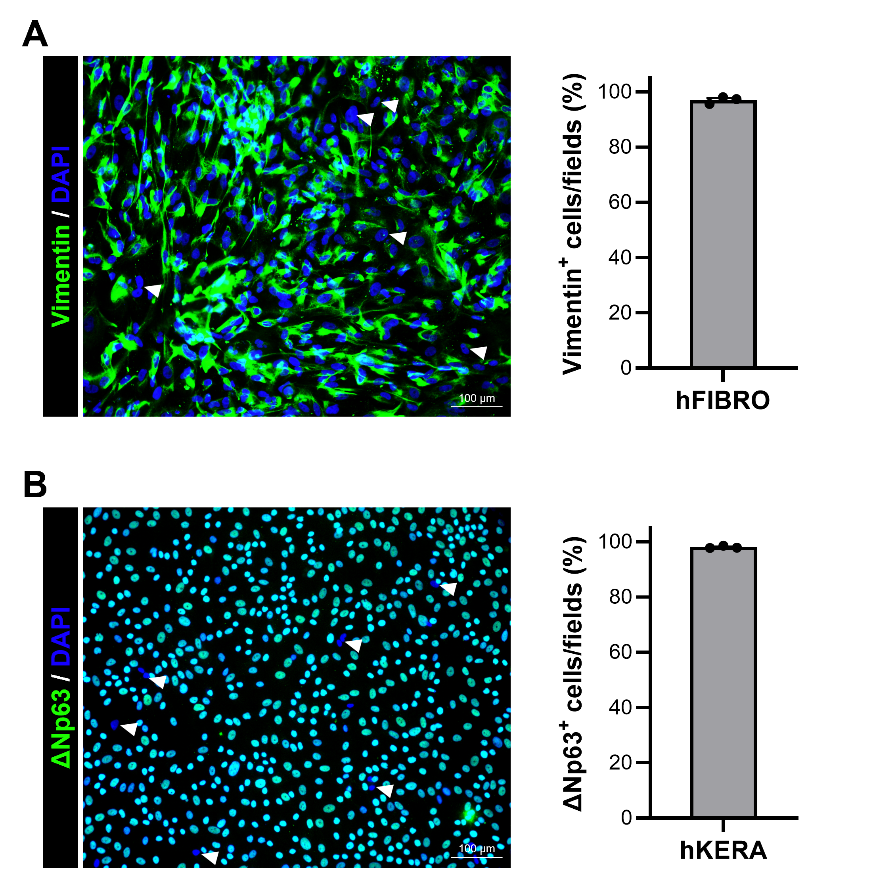


**Supplementary figure 1. IF staining for purity check in hFIBRO and hKERA.** (A) Staining for vimentin, a fibroblast-specific marker, in hFIBRO and distribution of vimentin^+^ cells per fields (n=3). (B) Staining for ΔNp63, a keratinocyte-specific marker, in hKERA and distribution of ΔNp63^+^ cells per fields (n=3). White arrows: DAPI where each marker is not expressed. Scale bar: 100 µm.


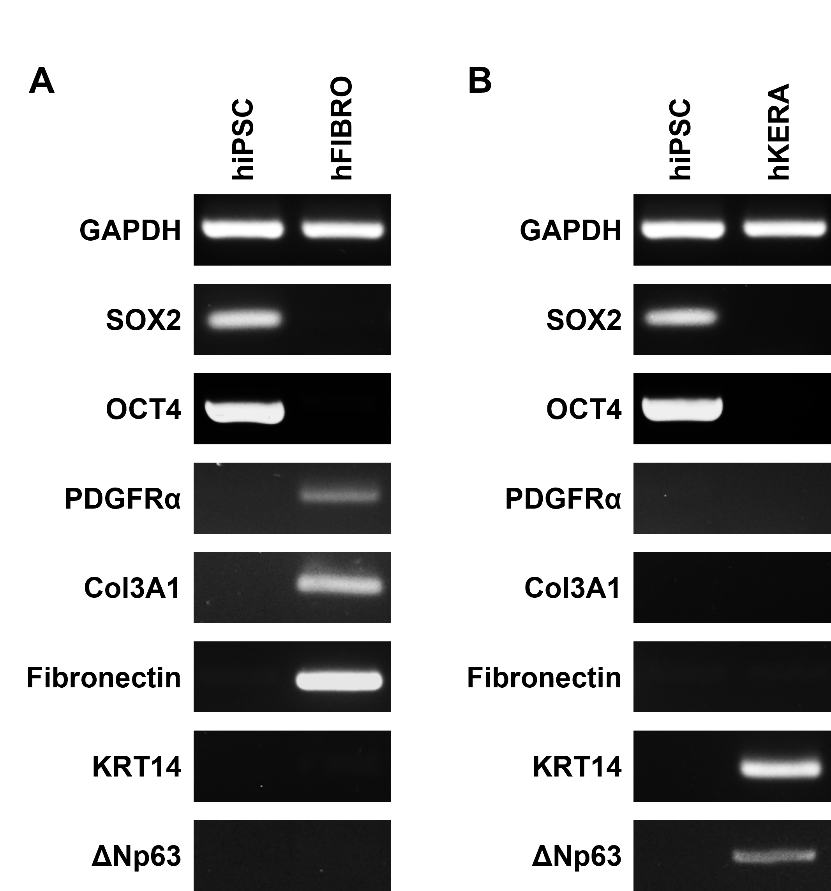


**Supplementary figure 2. Electrophoresis analysis for pluripotency and skin cell-specific markers by hiPSC, hFIBRO, and hKERA.** Genes: GAPDH (housekeeping gene for control), puripotency marekrs (OCT4 and SOX2), fibroblast-specific markers (PDGFRα, Col3A1, and Fibronectin), keratinocyte-specific markers (KRT14 and ΔNp63).
