# Supplementary material for: Skin irritation testing using human iPSCs derived 3D skin equivalent model
Source: PLoS One. 2025 Aug 18;20(8):e0330306. doi: 10.1371/journal.pone.0330306 (PMC12360594; doi:10.1371/journal.pone.0330306)
Supplement: Attachment — Submitted filename: Comments.docx [file pone.0330306.s002.docx]

The manuscript demonstrates a promising approach using hiPSC-derived cells to create a 3D skin model for irritation testing. However, there are significant areas that need improvement before it is ready for publication, primarily in the detail and clarity of the Materials and Methods and the Results sections, and in adding more quantitative data to support the conclusions. More concise writing and careful revision are necessary to enhance the clarity and improve the scientific rigor of the manuscript.

**Minor**

**Clarity and conciseness:** In some sections, the writing could benefit from improved clarity and conciseness. Some sentences are quite long and complex, making it difficult to follow the line of reasoning.

**Example 1 (Abstract)**

Original: "Research on artificial skin has advanced significantly, supporting cosmetic ingredient evaluation and skin regeneration treatment development. Artificial skin-based tests are well established, with standardized guidelines and widespread use of three-dimensional (3D) skin equivalent models (SKE) constructed from human primary skin cells. However, in the case of primary cells, there are problems with supply due to limited patients and difficulties in genetic-specific research."

Revised: "Advances in artificial skin research support cosmetic ingredient evaluation and skin regeneration. While 3D skin equivalent models (SKES) from human primary cells are established, limitations exist due to donor availability and difficulties in genetic-specific studies."

**Example 2 (Introduction):**

Original: "Among the various applications of SKEs, skin irritation and corrosion testing are prominent (7-9). International guidelines for skin irritation testing are well-established, with the Organization for Economic Cooperation and Development test guideline 439 (OECD TG 439) serving as a key reference (7-9). This testing is critical for verifying the safety of products such as cosmetics, pharmaceuticals, and chemicals by evaluating whether a substance causes physical or chemical irritation or damage to the skin. And OECD TG 439 proposes a method that uses an artificial skin model to replace traditional animal testing."

Revised: "SKEs are widely used for skin irritation and corrosion testing, guided by international standards like OECD TG 439. This testing is crucial for evaluating the safety of cosmetics, pharmaceuticals, and chemicals, and OECD TG 439 promotes artificial skin models as a replacement for animal testing."

**Specific questions**

**Cell purity and characterization:** The authors claim high purity for the differentiated hFIBROs and hKERAs. What specific methods beyond FACS analysis were used to confirm the purity and absence of undifferentiated hiPSCs? Could you provide data on the percentage of cells expressing pluripotency markers in the final populations?

**3D skin equivalent model (hiPSC-SKE) construction:** The protocol for hiPSC-SKE construction involves several steps. Could you explain in more detail the rationale behind the choice of specific media and growth factors at each stage? What optimization experiments were performed to determine the optimal cell densities and culture conditions for each layer?

**Triton X-100 as a positive control:** Why was Triton X-100 chosen as the positive control for skin irritation? Are there other commonly used positive controls, and why was this one preferred? Were there any considerations regarding potential non-specific effects of Triton X-100 on cell viability?

**Statistical analysis:** The statistical analysis uses one-way ANOVA. Are there post-hoc tests used to determine which groups are significantly different from each other? A more detailed explanation of the statistical methods is warranted.

**Mechanism of Triton X-100-induced damage:** The study shows that Triton X-100 causes damage to the epidermis. However, what are the mechanisms underlying this damage? Additional investigation into cell death pathways and signaling is needed.

**Methods section:** The methods section could benefit from more detailed information regarding the specific concentrations of reagents used, the duration of each treatment step, and the criteria used for assessing the success of the differentiation protocols.

**hiPSC generation:** The protocol for generating hiPSCs from human dermal fibroblasts is briefly described. Specify the episomal vectors used, the duration of reprogramming, and the selection methods used to enrich for hiPSC colonies.

**Differentiation protocols:** The protocols for differentiating hiPSCs into hFIBROs and hKERAs are incomplete. Specify the exact composition and concentrations of all growth factors and supplements in the various media (FDM1, FDM2, FDM3, KDM1, KDM2). Provide details on the cell culture conditions (temperature, CO2 concentration, etc.).

hiPSC-SKE construction: Describe the process of creating the hiPSC-SKE model in more detail. What type and concentration of collagen was used? What was the seeding density of the hKERAs? Were any additional factors used to promote keratinization?

Immunofluorescence: Clarify the antibodies used (company, catalog numbers). What was the counterstain used?

**Results**

The results are presented in a descriptive manner; some sections may be improved with more concise writing and more direct presentation of the findings.

The results section needs more quantitative data. Present specific percentages of cells positive for the different markers (vimentin, fibronectin, KRT14, ANp63) in the FACS and immunofluorescence analyses. Quantify the "significant morphological changes" observed in the hiPSC-SKE. Include numerical data on the thickness of the epidermal layers at different time points.

The figure legends provide some descriptive information, but more quantitative data should be incorporated where applicable. For example, specific measurements of epidermal thickness in Figure 2 should be included. The legends for Figures 3 and 4 would be improved by specifying the antibodies used and the types of staining techniques.

**Discussion:** The discussion section could be strengthened by including a more detailed comparison to other relevant research and a more thorough discussion of the potential limitations of the study. **The clinical implications of the findings should also be more explicitly stated.**

Comparison to existing SKEs: How do the characteristics of the hiPSC-SKE compare to commercially available SKEs (e.g., EpiDerm, Episkin) in terms of morphology, marker expression, and response to irritation testing?

Limitations and future directions: The authors mention limitations of using primary cells. What are the limitations of the hiPSC-SKE model presented in this study? What are the next steps or future directions of this research to overcome these limitations?
