# Supplementary material for: Skin irritation testing using human iPSCs derived 3D skin equivalent model
Source: PLoS One. 2025 Aug 18;20(8):e0330306. doi: 10.1371/journal.pone.0330306 (PMC12360594; doi:10.1371/journal.pone.0330306)
Supplement: Attachment — Submitted filename: Response to reviewer_PLOS ONE.docx [file pone.0330306.s003.docx]

PONE-D-25-01505

Skin Irritation Testing using Human iPSCs derived 3D Skin Equivalent Model

PLOS ONE

Dear Dr. Chung,

Thank you for submitting your manuscript to PLOS ONE. After careful consideration, we feel that it has merit but does not fully meet PLOS ONE’s publication criteria as it currently stands. Therefore, we invite you to submit a revised version of the manuscript that addresses the points raised during the review process.

1) I am not confident that we have a good quality of 3D skin. As pointed out by one of the reviewers, "There is no stratum granulosum and no stratum corneum formed. Differences in stratum basale and spinosum in H&E staining are only minor";

2) Please, see the comments raised by both the reviewers.

Please submit your revised manuscript by May 09 2025 11:59PM. If you will need more time than this to complete your revisions, please reply to this message or contact the journal office at [plosone@plos.org](mailto:plosone@plos.org). When you're ready to submit your revision, log on to https://www.editorialmanager.com/pone/ and select the 'Submissions Needing Revision' folder to locate your manuscript file.

Please include the following items when submitting your revised manuscript:

- A rebuttal letter that responds to each point raised by the academic editor and reviewer(s). You should upload this letter as a separate file labeled 'Response to Reviewers'.
- A marked-up copy of your manuscript that highlights changes made to the original version. You should upload this as a separate file labeled 'Revised Manuscript with Track Changes'.
- An unmarked version of your revised paper without tracked changes. You should upload this as a separate file labeled 'Manuscript'.

If you would like to make changes to your financial disclosure, please include your updated statement in your cover letter. Guidelines for resubmitting your figure files are available below the reviewer comments at the end of this letter.

If applicable, we recommend that you deposit your laboratory protocols in protocols.io to enhance the reproducibility of your results. Protocols.io assigns your protocol its own identifier (DOI) so that it can be cited independently in the future. For instructions see: <https://journals.plos.org/plosone/s/submission-guidelines#loc-laboratory-protocols>. Additionally, PLOS ONE offers an option for publishing peer-reviewed Lab Protocol articles, which describe protocols hosted on protocols.io. Read more information on sharing protocols at <https://plos.org/protocols?utm_medium=editorial-email&utm_source=authorletters&utm_campaign=protocols>.

We look forward to receiving your revised manuscript.

Kind regards,

Paulo Lee Ho, Ph.D.
Academic Editor
PLOS ONE

**Response to Editor:**

We sincerely thank the Editor’s genuine interest in our study and are grateful for permitting this revision of our manuscript. We did our best to experiment and consider in response to reviewers' comments. We hope that this revision coupled with our responses, are able to sufficiently address the critiques so our information and experience may be shared with the readership of PLOS ONE.

**Correction part**

**<Manuscript>**

**1) Abstract**

1. Research on artificial skin has advanced significantly, supporting cosmetic ingredient evaluation and skin regeneration treatment development. Artificial skin-based tests are well established, with standardized guidelines and widespread use of three-dimensional (3D) skin equivalent models (SKE) constructed from human primary skin cells. However, in the case of primary cells, there are problems with supply due to limited patients and difficulties in genetic-specific research. To address these issues, recent efforts have focused on differentiating skin cells from human-induced pluripotent stem cells (hiPSCs). 🡪 Artificial skin models have emerged as valuable tools for evaluating cosmetic ingredients and developing treatments for skin regeneration. Among them, 3D skin equivalent models (SKEs) using human primary skin cells are widely utilized and supported by standardized testing guidelines. However, primary cells face limitations such as restricted donor availability and challenges in conducting genotype-specific studies. To overcome these issues, recent approaches have focused on differentiating skin cells from human-induced pluripotent stem cells (hiPSCs).

2. The resulting hiPSC-SKE model replicated the primary layers of native skin morphologically, as demonstrated by hematoxylin and eosin staining, and expressed specific markers characteristic of each layer. Furthermore, treatment of hiPSC-SKEs with Triton X-100, a positive control for skin irritation testing, caused severe epidermal cell damage and reduced cell viability. These findings suggest that hiPSC-SKE serves as a promising alternative for skin-related applications, including animal testing replacement and genetic skin disease modeling. 🡪 Histological analysis with hematoxylin and eosin staining confirmed that the hiPSC-SKE recapitulated the layered architecture of native human skin and expressed appropriate epidermal and dermal markers. Moreover, exposure to Triton X-100, a known skin irritant, led to marked epidermal damage and significantly reduced cell viability, validating the model’s functional responsiveness. These findings indicate that the hiPSC-SKE model represents a promising alternative for various skin-related applications, including the replacement of animal testing.

**2) Introduction**

1. The skin is the primary tissue that exists on the body's exterior and performs various physiological functions in response to external environmental exposure (1, 2). 🡪 The skin, as the body's outermost tissue, serves as a barrier against environmental stimuli and performs essential physiological functions (1, 2).

2. Numerous studies have focused on creating human 3D skin equivalents (SKEs) using primary skin cells to replicate human skin in vitro (3-5). One advantage of SKEs is their relatively simple production through insert-based methods (6). In addition, when cultured keratinocytes on the surface in contact with the air forms epidermal-like layer, it forms an environment and structural complexity similar to human skin. 🡪 To replicate skin in vitro, 3D skin equivalent models (SKEs) have been developed using primary skin cells (3-5). SKEs can be fabricated relatively easily using insert-based methods (6). When keratinocytes are cultured on a surface exposed to air, they form the epidermis-like layer, creating an environment and structural complexity similar to that of human skin.

3. As a result, SKE has been used in various experiments related to skin drug toxicity evaluation, disease modeling, and transplantation therapeutics as wound models (5). 🡪 Due to these features, SKEs are widely used in studies related to drug toxicity, disease modeling, and wound healing applications (5).

4. Among the various applications of SKEs, skin irritation and corrosion testing are prominent (7-9). International guidelines for skin irritation testing are well-established, with the Organization for Economic Cooperation and Development test guideline 439 (OECD TG 439) serving as a key reference (7-9). This testing is critical for verifying the safety of products such as cosmetics, pharmaceuticals, and chemicals by evaluating whether a substance causes physical or chemical irritation or damage to the skin. And OECD TG 439 proposes a method that uses an artificial skin model to replace traditional animal testing. 🡪 SKEs are widely used for skin irritation and corrosion testing, guided by international standards like the Organization for Economic Cooperation and Development test guideline 439 (OECD TG 439). This testing is crucial for evaluating the safety of cosmetics, pharmaceuticals, and chemicals, and OECD TG 439 promotes artificial skin models as a replacement for animal testing (7-9).

5. Artificial skin used according to these guidelines typically includes Reconstructed Human Epidermis (such as RhE; EpiskinTM, EpiDermTM, SkinEthicTM RHE) and Dermo-Epidermal SKEs created from commercial primary skin cells (7-9). In addition to safety evaluations, keratinocytes and fibroblasts derived from human tissues are commonly used in research on skin diseases and therapeutic agents (6, 12-15). While primary cells reflect in vivo maturity, challenges such as limited supply, high cost, and senescence from passaging remain. Additionally, simulating genetic disease models is difficult due to limitations in manufacturing patient-specific products, and immune rejection complicates therapeutic agent transplantation. To address these issues, human induced pluripotent stem cells (hiPSCs), known for their self-renewal and pluripotency, have emerged as a promising source (16, 17). The advantages of hiPSC-derived cells include: 1) overcoming cell supply constraints through self-renewal, 2) disease modeling using patient-derived genetic samples or gene editing, and 3) superior therapeutic effects through greater engraftment with low immunogenicity (16, 17). Some studies have successfully produced 3D SKEs from hiPSC-derived skin cells (18-21). However, reports on the production and use of hiPSC-derived SKEs (hiPSC-SKEs) are still limited, and creating a comprehensive database of studies is essential to refine these models. In this study, we established a protocol for producing skin cells from hiPSCs and successfully generated hiPSC-SKEs. Morphological analysis confirmed that hiPSC-SKEs replicate human skin characteristics, and skin irritation tests using Triton X-100 were successfully conducted. These databases are expected to play a significant role in fields such as medicine, cosmetics, and drug development, as hiPSC-SKEs evolve into more advanced skin models in the future. 🡪 Artificial skin models used according to established guidelines typically include reconstructed human epidermis (RhE) products such as Episkin™, EpiDerm™, and SkinEthic™ RHE, as well as dermo-epidermal SKEs constructed from commercially available primary skin cells  (7-9). In addition to safety evaluations, skin cells are commonly used in research on skin diseases and therapeutic agents (6, 12-15). While primary cells reflect in vivo maturity, their use is limited by supply shortages, high costs, and senescence during passaging. Additionally, modeling genetic skin diseases is challenging due to difficulties in producing patient-specific products, and immune rejection complicates therapeutic transplantation. To address these issues, human induced pluripotent stem cells (hiPSCs) have emerged as a promising source (16, 17). Advantages of hiPSC-derived cells include: (1) unlimited supply through self-renewal, (2) disease modeling via patient-specific genetics or gene editing, and (3) improved therapeutic potential with better engraftment and low immunogenicity (16, 17). Several studies have successfully generated 3D SKEs from hiPSC-derived skin cells (18-21). However, reports on hiPSC-derived SKEs (hiPSC-SKEs) remain limited, and a comprehensive study database is needed to optimize these models. In this study, we established a protocol to differentiate skin cells from hiPSCs and generated hiPSC-SKEs. Morphological analyses confirmed that hiPSC-SKEs replicate human skin features, and skin irritation tests using Triton X-100 were successfully performed. These datasets are expected to contribute significantly to cosmetics, chemicals, and drug development as hiPSC-SKEs advance as skin models.

**3) Materials & Methods**

1. Added sentence ‘(Generation of human induced pluripotent stem cells (hiPSCs)) 1.5 × 10⁶ human dermal fibroblasts (BJ cells; ATCC, VA, USA) were transfected with episomal vectors (pCLXE-hOCT4/p53, pCLXE-hSOX2/KLF4, and pCLXE-hL-MYC/LIN28A; Addgene, MA, USA) using the P2 Primary Cell 4D-Nucleofector Kit (Lonza, Basel, Switzerland) according to the manufacturer’s protocol (22, 23). The mixture of cells and vectors was transferred into a Nucleocuvette Vessel and subjected to electroporation. Transfected cells were plated onto 1:100 Matrigel (Corning, NY, USA)-coated 6-well plates and cultured for 2 days in Dulbecco’s Modified Eagle’s Medium (DMEM; Gibco, NY, USA) supplemented with 10% fetal bovine serum (FBS; Sigma-Aldrich, St. Louis, MO, USA), 0.1 mM non-essential amino acids (NEAA; Gibco), and 1% penicillin/streptomycin (P/S; Gibco). After 2 days, the medium was replaced with TeSR-E7 medium (STEMCELL Technologies, Vancouver, Canada), which was changed daily. Cells were cultured for an additional 10–14 days until PSC-like colonies appeared. After picking the PSC-like colonies, they were seeded onto Matrigel-coated 4-well plates in mTeSR1 (STEMCELL Technologies Inc; Vancouver, BC, Canada) medium supplemented with 10 µM Y-27632 (Tocris Bioscience, Bristol, UK). The medium was changed daily with fresh mTeSR1 during subsequent culture.’.

2. hiPSCs were produced using a pluripotency-related reprogramming episomal vectors (addgene, MA, USA) in human dermal fibroblasts (ATCC, VA, USA) (22, 23). hiPSCs were seeded on 60 mm cell culture dish coated with 1:100 Matrigel (Corning Inc.; Corning, NY, USA) and cultured by replacing the mTeSR1 (STEMCELL Technologies Inc; Vancouver, BC, Canada) medium every day. After 3 days, when 90-100% confluency was achieved, hiPSCs were detached using DPBS (welgene, Gyeongsan, South Korea) containing 0.5 mM EDTA (Gibco, NY, USA). Detached hiPSCs were collected in 6 ml mTeSR1 containing 10 µM Y-27632 (Tocris Bioscience, Bristol, UK) and placed in a 60 mm petri dish coated with F127 (Sigma-Aldrich, St. Louis, USA). Afterwards, they were cultured for one day in a shaker set at 65-70 rpm in a 37 °C, 5% CO2 incubator. The next day, embryonic bodies (EBs) formation was confirmed and mTeSR1 was replaced every day for 2 days (D3). 150-200 EBs were seeded on Matrigel-coated 60 mm cell culture dishes using fibroblast differentiation media 1 (FDM1) and replaced for 3 days. FDM1 is a 3:1 mixture of DMEM (Gibco, NY, USA) and F12 (Gibco) supplemented with 5% fetal bovine serum (FBS; Sigma-Aldrich), 5 µg/mL Insulin (Sigma-Aldrich) and 10 ng/ml epithelial growth factor (EGF; PeproTech, NJ, USA). After 3 days, FDM2 was replaced and cultured for 3 days. FDM2 is a medium supplemented with 25 ng/ml bone morphogenic protein 4 (BMP4; Peprotech) to FDM1. Next, it was cultured for 7 days after replacing with FDM3. FDM3 is a 1:1 mixture of DMEM and F12 supplemented with 1% non-essential amino acids solution (NEAA; Gibco). After 7 days, it was replaced with FDM1 medium again and cultured for 4 days. When it reached 70-80% confluency, it was detached using a 5:1 mixture of TrypLE (Gibco) and 0.25% Trypsin/EDTA (T/E; Gibco) (D20). The detached cells were seeded on matrigel-coated cell culture dishes with FDM1 medium and maintained in culture until D30 (Fig. 1A). 🡪 hiPSCs were seeded onto 1:100 Matrigel-coated 60 mm cell culture dishes and maintained in mTeSR1 medium with daily medium changes. Upon reaching 90–100% confluency after 3 days, the cells were gently dissociated using DPBS (Welgene, Gyeongsan, South Korea) containing 0.5 mM EDTA (Gibco). The detached hiPSCs were resuspended in 6 ml mTeSR1 supplemented with 10 µM Y-27632 and transferred to F127-coated (Sigma-Aldrich) 60 mm Petri dishes to initiate embryoid body (EB) formation (D0). Cells were cultured on a shaker at 65–70 rpm in a 37 °C incubator with 5% CO₂ for 24 hr. On the following day (D1), EB formation was confirmed, and mTeSR1 was replaced daily for two additional days (until D3). Approximately 150–200 EBs were then transferred onto Matrigel-coated 60 mm culture dishes and cultured in fibroblast differentiation medium 1 (FDM1) for 3 days (D3-D6). FDM1 consisted of a 3:1 mixture of DMEM (Gibco) and F12 (Gibco), supplemented with 5% FBS, 5 µg/ml insulin (Sigma-Aldrich), and 10 ng/ml epidermal growth factor (EGF; PeproTech, NJ, USA). After 3 days, the medium was changed to fibroblast differentiation medium 2 (FDM2), which is FDM1 supplemented with 25 ng/ml bone morphogenetic protein 4 (BMP4; PeproTech), and cultured for another 3 days (D6-D9). Subsequently, the cells were cultured in fibroblast differentiation medium 3 (FDM3) for 7 days (D9-D16). FDM3 consisted of a 1:1 mixture of DMEM and F12 supplemented with 5% FBS and 1% NEAA. Upon reaching 70–80% confluency (Day 16), cells were dissociated using a 5:1 mixture of TrypLE (Gibco) and 0.25% Trypsin-EDTA (Gibco). The detached cells were replated on Matrigel-coated dishes in FDM1 and maintained until Day 30 (D30) (Fig. 1A).

3. 70-80 EBs were seeded on Matrigel-coated 60 mm cell culture dishes using keratinocyte differentiation media 1 (KDM1) and replaced for 7 days. KDM1 is a 3:1 mixture of DMEM and F12 supplemented with 2% FBS, 5 µg/mL Insulin, 25 ng/ml EGF, 25 ng/ml BMP4 and 1 µg/mL Retinoic acid (RA; Sigma-Aldrich). After 7 days, KDM2 was replaced and cultured for 7 days. KDM2 is a medium supplemented with 0.5% FBS, 5 µg/mL Insulin, 20 ng/ml EGF, 20 ng/ml BMP4 and 1 µg/mL RA to Defined Keratinocyte serum-free medium (DKSFM; Gibco). Then, it was cultured for 7 days after replacing CnT-07 medium (CELLnTEC, Bern, Switzerland). When it reached 70-80% confluency, it was detached using a 5:1 mixture of TrypLE and 0.25% T/E (D24). The detached cells were seeded on matrigel-coated cell culture dishes with CnT-07 medium and maintained in culture until D30 (Figure 1E). 🡪 70-80 EBs were seeded onto Matrigel-coated 60 mm cell culture dishes and cultured in keratinocyte differentiation medium 1 (KDM1) for 7 days, with daily medium changes (D3-D10). KDM1 consisted of a 3:1 mixture of DMEM and F12, supplemented with 2% FBS, 5 µg/mL insulin, 25 ng/ml EGF, 25 ng/ml BMP4, and 1 µg/ml retinoic acid (RA; Sigma-Aldrich). After 7 days, the medium was replaced with keratinocyte differentiation medium 2 (KDM2) and cells were cultured for an additional 7 days (D10-D17). KDM2 consisted of Defined Keratinocyte Serum-Free Medium (DKSFM; Gibco) supplemented with 0.5% FBS, 5 µg/ml insulin, 20 ng/ml EGF, 20 ng/ml BMP4, and 1 µg/ml RA. Subsequently, the cells were cultured for 7 days in CnT-07 medium (CELLnTEC, Bern, Switzerland) (D17-D24). When cells reached 70–80% confluency (D24), they were dissociated using a 5:1 mixture of TrypLE (Gibco) and 0.25% Trypsin-EDTA (Gibco). The detached cells were replated on Matrigel-coated culture dishes and maintained in CnT-07 medium until Day 30 (D30) (Figure 1E).

4. Mixture of 4.5 mg/ml Collagen type Ⅰ (Corning) and 6 x 104 hFIBROs was poured into pore polyester membrane insert (Corning) to create dermis. After clotting in a 37 ℃, 5% CO_2 for 30 min, it was cultured by replacing FDM1 medium every day. After 3 days, 1.5 x 106 hKERAs were spread on the dermis with Cnt-07 medium, and the medium was changed every day for 3 days. Then, it was cultured for 4 days after replacing CnT-airlift medium (CELLnTEC) containing 1 mM Calcium Chloride (Promocell, Heidelberg, Germany). Next, the medium was completely removed from the insert to induce keratinization, and air-liquid culture was performed until D30. 🡪 To construct the dermal layer, a mixture of 4.5 mg/mL collagen type I (Corning, NY, USA, Cat# 354249) and 6 × 10⁴ hFIBROs was poured into a porous polyester membrane insert (Corning) and allowed to solidify in a 37 °C incubator with 5% CO₂ for 30 min. Then, the construct was cultured in FDM1 medium with daily medium changes for 3 days (D0-D3). Subsequently, 1.5 × 10⁶ hKERAs were seeded onto the surface of the dermal layer in CnT-07 medium, which was replaced daily for 3 days (D3-D6). The culture was then switched to CnT-airlift medium (CELLnTEC) supplemented with 1 mM calcium chloride (CaCl2; PromoCell, Heidelberg, Germany) and maintained for an additional 4 days (D6-D10). To induce epidermal keratinization, the medium was completely removed from the insert, and an air–liquid culture was maintained for 20 days (D10-D30). CnT-airlift medium was added beneath the insert and replaced every other day (24).

5. Added catalog numbers ‘PE-conjugated Mouse Anti-Human Vimentin (BD Biosciences; CA, USA, Cat# 562337; 1:25)’ and ‘FITC-conjugated Anti-Cytokeratin 14 antibody (KRT14; abcam, Cambridge, England, Cat# ab77684; 1:25)’.

6. As primary antibodies, anti-Vimentin (abcam; 1:100, ab8978), anti-Fibronectin (Fibronectin; 1:100, ab2413) were used for hFIBROs and anti-ΔNp63 (abcam; 1:100, ab203826), anti-keratin 14 (KRT14; abcam; 1:100, ab181595) were used for hKERAs staining. 🡪 As primary antibodies, anti-Vimentin (abcam, Cat# ab8978; 1:200), anti-Fibronectin (abcam, Cat# ab2413; 1:200), anti-ΔNp63 (abcam, Cat#203826; 1:100), and anti-keratin 14 (KRT14; abcam, Cat# ab181595; 1:200) were used.

7. Next, it was washed twice with PBS for 15 min each and treated with DAPI (Thermo Fisher Scientific, MA, USA; 1:1000) for 1 min. 🡪 Next, it was washed twice with PBS for 15 min each and treated with DAPI (4′,6-diamidino-2-phenylindole; Sigma-Aldrich, Cat# D9542) for 1 min. DAPI was used as the nuclear counterstain.

8. As primary antibodies, anti-ΔNp63, anti-KRT14, anti-Vimentin, anti- platelet-derived growth factor receptor α (PDGFRα; abcam, ab32570), and anti-Fibronectin were used for 1:200. 🡪 As primary antibodies, anti-Vimentin (abcam, Cat# ab8978; 1:200), anti-Fibronectin (abcam, Cat# ab2413; 1:200), anti-ΔNp63 (abcam, Cat#203826; 1:100), anti-KRT14 (abcam, Cat# ab181595; 1:200), anti-platelet-derived growth factor receptor α (PDGFRα; abcam, ab32570), anti-KRT10 (abcam, Cat# ab76318; 1:200), and anti-Loricirin (abcam, Cat# ab198994; 1:100) were used.

9. Added sentence ‘(Gel electrophoresis) To analyze the gene expression in hFIBROs and hKERAs, cells were harvested on day 30 of differentiation. Total RNA was isolated using TRIzol reagent (Invitrogen, Grand Island, NY, USA) following the manufacturer’s instructions. 1 μg RNA was used for cDNA synthesis with AccuPower RT PreMix and Oligo(dT) primers (Bioneer, Daejeon, Korea). The synthesized cDNA was then mixed with gene-specific primers and AccuPower® PCR PreMix (Supple table. 1). PCR was performed and the presence of target gene bands was verified for each sample by electrophoresis. The information of primers was described in Supplementary Table 1. Also, we used GAPDH as PCR control.’.

10. Data were presented as mean ± SEM, and the statistical significance of the experimental results was calculated using one-way ANOVA. 🡪 Data were presented as mean ± SEM, and the statistical significance of the experimental results was calculated using either a T-test or one-way ANOVA with Tukey’s method for comparison between groups.

**4) Results**

1. To generate fibroblasts, the principal cellular components of the dermis, from human induced pluripotent stem cells (hiPSCs), we established a protocol based on cytokines and small molecules (Fig. 1A). The morphology of hiPSC-derived fibroblasts (hFIBROs) changed as differentiation progressed (Fig. 1B). Embryoid bodies (EBs) attached to Matrigel-coated dishes proliferated and adopted a mesenchymal morphology similar to that of fibroblasts starting from day 16 (D16). Even after subculturing, the differentiated hFIBROs maintained their mesenchymal morphology at day 30 (D30) (Fig. 1B). 🡪 To generate human induced pluripotent stem cells (hiPSCs) derived fibroblasts (hFIBROs), we established a protocol based on cytokines and small molecules by referring to previous reports (Fig. 1A) (21, 25). During differentiation, morphological changes consistent with mesenchymal transition were observed. Embryoid bodies (EBs) plated onto Matrigel-coated dishes proliferated and began adopting a spindle-shaped, fibroblast-like morphology by day 16 (D16), which was maintained after passaging up to day 30 (D30) (Fig. 1B).

2. Added sentence ‘The purity of hFIBROs was assessed by analyzing the density of vimentin+ cells per field, which was 97.01 ± 0.73% (Supple fig. 1A).’.

3. FACS analysis results showed that Vimentin+ cells were 92.94%, which was consistent with the IF results. (Fig. 1D). 🡪 Also, FACS analysis confirmed that 92.94% of the cells were vimentin+, consistent with IF staining. (Fig. 1D).

4. Next, we established a protocol for generating keratinocytes, the main cellular components of the epidermal layer, from hiPSCs (Fig. 1E). After seeding the EBs, a cobblestone morphology characteristic of keratinocytes was observed from day 17 (D17) as differentiation progressed. Differentiated hiPSC-derived keratinocytes (hKERAs) maintained this cobblestone morphology at D30, even as proliferation continued (Fig. 1F). 🡪 Next, we established a protocol for generating hiPSC derived keratinocytes (hKERAs) (Fig. 1E) (21, 24, 25). Following EB attachment, cells began to exhibit a cobblestone morphology characteristic of keratinocytes by day 17 (D17), which persisted through D30 (Fig. 1F).

5. Added sentence ‘The purity of hKERAs was assessed by analyzing the density of ΔNp63+ cells per field, which was 98.09 ± 0.24% (Supple fig. 1B).’.

6. FACS analysis showed that 97.48% of the cells were KRT14+ (Fig. 1H). 🡪 FACS analysis further confirmed the efficiency of differentiation, with 97.48% of cells expressing KRT14 (Fig. 1H).

7. Added sentence ‘To further confirm the absence of residual undifferentiated cells in the differentiated hFIBROs and hKERAs, gel electrophoresis analysis for pluripotency markers was performed (Supple fig. 2). Pluripotent markers such as SOX2 and OCT4 were strongly expressed in undifferentiated hiPSCs but were undetectable in both hFIBROs and hKERAs. In contrast, fibroblast-specific markers (PDGFRα, Col3A1, and fibronectin) were exclusively expressed in hFIBROs (Supple fig. 2A), while keratinocyte-specific markers (KRT14 and ΔNp63) were detected only in hKERAs (Supple fig. 2B).’.

8. Taken together, the established protocols successfully differentiated hFIBROs and hKERAs, which closely mimic primary skin cells with high purity.🡪 These results confirm successful lineage-specific differentiation and effective elimination of PSCs, demonstrating that the established protocols reliably generate hFIBROs and hKERAs.

9. Following this, hKERAs were seeded onto the constructed dermis to form a layer, and keratinization of the epidermis was induced using air-liquid interface (ALI) culture (Fig. 2A). As differentiation progressed, the upper surface of hiPSC-SKE exhibited changes; after ALI culture, the epidermal layer became uneven, with some edges contracting (Fig. 2B). Hematoxylin and eosin (H&E) staining was performed to evaluate morphological changes in the epidermis at the early (D10) and late (D20) stages of ALI culture (Fig. 2C). The epidermal layer was thicker on D20 than on D10, with the stratum basale (SB) and stratum spinosum (SS)-like layers beneath the stratum corneum (SC) also showing increased thickness (Fig. 2C) (13). Additionally, the morphological similarity of hiPSC-SKE to native skin (human and mouse) was assessed using H&E staining, confirming structural resemblance in the dermis and epidermis (Fig. 2D). 🡪 Subsequently, hKERAs were seeded onto the constructed dermis to form an epidermal layer, and keratinization was induced via air-liquid (ALI) culture (Figs. 2A and 2B). As differentiation progressed, the upper surface of hiPSC-SKE exhibited changes. After ALI culture, the epidermal layer became uneven, with some edges contracting. (Fig. 2B). H&E staining was performed to evaluate epidermal morphology at early (D20; ALI culture for 10 days) and late (D30; ALI culture for 20 days) stages of ALI culture (Fig. 2C). Keratinization of the epidermis was more prominent at ALI-20 days than at ALI-10 days of ALI culture. The epidermal thickness was significantly increased at ALI-20 days (ALI-10 days: 38.82 ± 2.20 μm vs. ALI-20 days: 63.62 ± 8.06 μm), accompanied by thickening of the stratum basale (SB) and stratum spinosum (SS)-like layers. Furthermore, a stratum corneum (SC)-like structure was observed at the air-exposed surface at ALI-20 days (Figs. 2C and 2D) (13). Comparative H&E analysis revealed that both the dermal and epidermal layers of hiPSC-SKE closely resemble those of native human and mouse skin in structure (Fig. 2E).

10. To examine the distribution of constituent cells and ECM within the major layers of hiPSC-SKE, IF staining was conducted (Fig. 3). The distribution of hKERAs within the epidermis was confirmed using keratinocyte-specific markers (ΔNP63 and KRT14). These markers were expressed in the SB-like layer of the epidermis, adjacent to the dermis, where proliferating keratinocytes are predominantly located (Figs. 3A and 3B) (26, 29). 🡪 To investigate the progression of keratinization induced by ALI culture in the epidermis, representative markers of major epidermal layers were examined at 10 and 20 days of ALI culture. IF staining for KRT14 and ΔNp63, representative markers of keratinocytes predominantly expressed in the SB, showed strong expression in the lower part of the epidermis (Figs. 3A and 3B). Notably, ΔNp63 expression gradually decreased toward the upper layers at ALI-20 days (D30), compared to ALI-10 days (D20) (Fig. 3B). KRT10, a marker primarily expressed in the SS, was strongly detected above the SB, and its expression domain became thicker at ALI-20 days compared to ALI-10 days (Fig. 3C). Furthermore, loricrin, a late differentiation marker expressed in the SC, was not detected at ALI-10 days but was clearly expressed in a band-like pattern at ALI-20 days (Fig. 4D) (20, 28, 31).

11. Skin irritation testing is a safety evaluation method used in the development of new drugs and cosmetics, assessing tissue damage such as cell death caused by exposure of artificial skin to a substance (Fig. 4A) (30). In this test, sodium dodecyl sulfate (SDS) or Triton X-100 serves as the positive control, and an absorbance-based cell viability test is conducted to evaluate the extent of tissue damage. In this study, hiPSC-SKEs were treated with 3% Triton X-100 (3% Triton), and the CCK-8 assay was used to assess cell viability rate. Triton X-100 is a nonionic detergent that binds specifically to lipids, permeabilizes cell membranes, and induces cell death (31). 🡪 Skin irritation testing is a type of alternative animal testing that evaluates cell damage in artificial skin models following exposure to chemical substances (Fig. 4A). In this test, sodium dodecyl sulfate (SDS) or Triton X-100, which are known to cause cytotoxicity as a side effect, are used as a positive control, and the degree of tissue damage is assessed through an absorbance-based cell viability test (32). To evaluate the utility of hiPSC-SKEs in skin irritation testing, a CCK-8 based cell viability assay using 3% Triton X-100 was conducted. Triton X-100 is a nonionic detergent that disrupts cell membranes by specifically binding to membrane lipids, damaging the structural integrity of the membrane. This increases membrane permeability and causes ion imbalance, ultimately disrupting cellular homeostasis and inducing cell death. Additionally, increased cellular stress damages the mitochondrial membrane potential, further promoting cell death (33-36). Triton X-100 induces reproducible skin irritation under standardized conditions with minimal nonspecific cytotoxicity, making it a widely used positive control in skin irritation testing.

12. To ensure that the treatment drug penetrated the skin, 3% Triton was applied inside the insert containing the hiPSC-SKEs, while only the basic medium was placed in the outer well. For comparison, culture medium (CnT-Prime Airlift) was used as the negative control (Control). After 1.5 hr of treatment, washing was performed, and the post-treatment (Post-treat) O.D. value was measured using the CCK-8 assay (Fig. 4B). WST-8 in the CCK-8 reagent is reduced by mitochondrial dehydrogenase to form orange formazan (32). Similar color changes were observed between the control and the 3% Triton pre-treat (Fig. 4C), with no significant difference in the O.D. value at 450 nm (Fig. 4D). However, while the control group showed no color change from pre- to post-treat, the 3% Triton group exhibited a faint orange color (Fig. 4C). Furthermore, the O.D. value was significantly reduced in the 3% Triton group (pre-treat: 1.053 ± 0.158, post-treat: 0.045 ± 0.002), in contrast to the control group (pre-treat: 1.336 ± 0.109, post-treat: 1.272 ± 0.179) (Fig. 4D). These results indicate that the viability of hiPSC-SKEs decreased as a result of cell death induced by 3% Triton. Additionally, the skin damage caused by 3% Triton was observed from a morphological perspective using H&E staining. Severe damage to the epidermal cells near the air interface was evident, distinguishing the 3% Triton-treated group from the control group (Fig. 4E). In summary, hiPSC-SKE has been proven to be a promising source for skin safety assessment of various skin irritation. 🡪 To ensure proper penetration of the treatment agent, 3% Triton was applied inside the insert containing the hiPSC-SKEs, while only basic medium was added to the outer well. As a negative control, culture medium (CnT-Prime Airlift) was used. After 1.5 hr of treatment, the hiPSC-SKEs were washed, and the post-treatment (post-treat) O.D. was measured again using the CCK-8 assay (Fig. 4B). In this assay, WST-8 is reduced by mitochondrial dehydrogenase to produce orange-colored formazan (37). As shown in Fig. 4C, similar color changes were observed between the control and 3% Triton groups at the pre-treat stage, with no significant differences in O.D. (Fig. 4D). However, after post-treat stage, the control group showed no change in color, whereas the 3% Triton group exhibited only a faint orange color (Fig. 4C). Correspondingly, a significant decrease in O.D. was observed in the 3% Triton group (pre-treat: 1.053 ± 0.158; post-treat: 0.045 ± 0.002), while the control group maintained relatively stable O.D. (pre-treat: 1.336 ± 0.109; post-treat: 1.272 ± 0.179) (Fig. 4D). These results suggest that 3% Triton induced cell death in hiPSC-SKEs, leading to reduced viability. Morphological analysis using H&E staining further confirmed severe damage to the epidermal layer near the air interface in the 3% Triton group, which was not observed in the control group (Fig. 4E). In summary, hiPSC-SKE has been proven to be a valuable model for skin safety assessment of various skin irritation.

**5) Discussion**

1. Our study showed that hiPSC-derived SKE can serve as a viable alternative to existing primary skin cell-based SKE and highlighted the potential applications of hiPSC-SKE in skin-related research. hiPSC-SKE was generated by differentiating hiPSC-derived fibroblasts and keratinocytes. The formation of skin composition layers resembling native skin was confirmed, along with the cellular distribution within each layer. Additionally, treating hiPSC-SKEs with 3% Triton and conducting cell viability and morphological analyses suggested its applicability in skin irritation testing, a recognized alternative to animal testing. 🡪 Our study demonstrated that hiPSC-derived skin equivalents (hiPSC-SKEs) can serve as a viable alternative to conventional primary skin cell-based models. hiPSC-SKE was generated by differentiating hiPSC-derived fibroblasts and keratinocytes. The formation of skin composition layers resembling native skin was confirmed, along with the cellular distribution within each layer. Furthermore, treatment of hiPSC-SKEs with 3% Triton X-100, followed by assessments of cell viability and morphological changes, supported their potential utility in skin irritation testing.

2. Previous studies have reported the development of SKE models using hiPSC-derived skin cells as replacements for primary skin cells. Some of these models involved a mixture of hiPSC-derived cells and primary skin cells (18, 20), while others were constructed by differentiating both fibroblasts and keratinocytes from hiPSCs (18, 19, 21). Comparing our findings with earlier studies revealed differences in SKE morphology, such as epidermal or SC thickness and dermal cell distribution, likely due to variations in cell density, culture duration, and medium composition. Despite these differences, most SKEs exhibited a similar patterns to native skin. The epidermis matures and ages through keratinization initiated in the SB, forming the sublayers SS, stratum granulosum (SG), and SC toward the air interface. 🡪 Previous studies have reported the development of SKEs using hiPSC-derived skin cells as substitutes for primary skin cells. Some of these models incorporated a mixture of hiPSC-derived and primary skin cells (18, 20), while others were constructed entirely from hiPSC-derived fibroblasts and keratinocytes (18, 19, 21). When compared to previous reports, our SKEs showed morphological differences—such as variations in epidermal and SC thickness and dermal cell distribution. These differences may result from factors such as cell density, culture period, and media composition. Despite these variations, most SKEs, including ours, exhibited structural patterns comparable to native human skin. Epidermal maturation in native skin proceeds through keratinization, starting from the SB and forming the SS, SG, and SC toward the air interface.

3. Our results confirmed that KRT14 and ΔNp63 were strongly expressed only in the area adjacent to the dermis in the keratinized epidermis through ALI culture in hiPSC-SKE, and that expression diminishing toward the air interface (Figs. 3A and 3B). This indicates that ΔNp63+ and KRT14+ cells were confined to the SB, with the suprabasal layers SS, SG, and SC lacking these markers (33). In native skin, SS expresses KRT1 and KRT10, SG expresses loricrin, and SC expresses filaggrin (33). 🡪 Consistent with this, our results confirmed that KRT14 and ΔNp63 were strongly expressed in the basal region adjacent to the dermis in hiPSC-SKEs generated by ALI culture, with expression levels gradually diminishing toward the surface (Figs. 3A and 3B) (38). In native skin, KRT1 and KRT10 are primarily expressed in the SS, while loricrin and filaggrin are mainly expressed in the SC (39). In our hiPSC-SKEs, we also demonstrated that the KRT10+ layer thickened as ALI culture progressed, and loricrin, which was not observed at ALI-10 days, became detectable by ALI-20 days (Figs. 3C and 3D).

4. Type IV collagen forms the primary structural component of the BM, creating a meshwork embedded with laminin and fibronectin (34). 🡪 Type IV collagen is the major structural component of the BM, forming a meshwork that incorporates laminin and fibronectin (40).

5. Skin irritants penetrate the SC and damage basal skin cells. Additionally, damaged skin cells trigger an inflammatory response in the dermis, leading to erythema and edema, which are key characteristics of tissue damage resulting from skin irritation. To evaluate this phenomenon in vitro, an alternative to animal testing has been developed using artificial skin, and extensive research findings have contributed to a database that now serves as a guideline. Skin irritation testing using SKEs assesses tissue damage by conducting cell viability assays, such as the MTT assay, following topical application of the test substance (7-9). According to the United Nations Globally Harmonized System of Classification and Labeling of Chemicals (UN GHS) criteria, if the average tissue viability after application of the test substance and post-treatment exceeds 50%, the substance is not considered an irritant. Conversely, if the viability is ≤50%, the substance is classified as an irritant, and additional information on skin corrosivity is required for more detailed classification. 🡪 Skin irritants penetrate the SC and damage basal skin cells, triggering an inflammatory response in the dermis that leads to erythema and edema, which are key signs of skin irritation. To evaluate this in vitro, artificial skin models have been developed as alternatives to animal testing, supported by a robust database that now serves as a guideline. Skin irritation testing using SKEs typically involves cell viability assays, such as the MTT assay, after topical application of test substances (7-9). According to the United Nations Globally Harmonized System of Classification and Labeling of Chemicals (UN GHS), a substance is classified as non-irritant if the average tissue viability after treatment exceeds 50%. In contrast, substances resulting in ≤50% viability are classified as irritants, and further assessment for skin corrosivity is required for detailed categorization.

6. These findings confirm that Triton X-100, a positive control for skin irritation in conventional primary skin cell-based SKE, also induces irritation in hiPSC-SKE. 🡪 These results confirm that Triton X-100, a well-established positive control for skin irritation in primary cell-based SKE models, also induces significant irritation in hiPSC-SKEs.

7. Although this study evaluated only one substance using hiPSC-SKE, the results suggest its potential applicability for assessing other substances. Future studies will include skin irritation tests for cosmetics and pharmaceuticals under development, as well as other skin irritants (e.g., cyclamen aldehyde, heptanal) specified in existing guidelines. Moreover, therapeutic effects have previously been demonstrated in a wound model using SKE based on primary skin cells and 3D bioprinting (15). The hiPSC-SKE, derived from PSCs with lower immune rejection responses, is anticipated to exhibit superior therapeutic effects (36-38). With our results, domestic and international databases are being accumulated, and it is expected that skin irritation test guidelines using hiPSC-SKE will be established in the future. In addition, hiPSC-SKE can be used in various skin-related research fields, such as customized treatment for skin wound patients and genetic skin disease modeling. 🡪 hiPSC-SKEs demonstrated marker expression and irritation responses comparable to primary skin cell-based SKEs, supporting their potential as reliable alternatives. hiPSCs offer advantages such as overcoming limitations in cell supply, cost, and donor variability. However, a key challenge remains as hiPSC-derived cells often exhibit an immature phenotype (42, 43). Similarly, hiPSC-derived skin organoids lack structural completeness, such as Collagen VII and immune or endothelial cells (44, 45). This immaturity may also affect hiPSC-derived skin cells, potentially explaining the need for ALI culture durations exceeding 20 days. Moreover, SKEs generated from primary skin cells often display a thicker SC compared to hiPSC-SKEs (20, 24). To enhance model maturity, future studies should incorporate additional skin constituent cells to better reflect in vivo conditions. Batch-to-batch variability during hiPSC differentiation also poses challenges for reproducibility. To address this, we are exploring previously reported droplet-based bioprinting and nebulization technologies to standardize hiPSC-SKE fabrication. In summary, the hiPSC-SKE model offers a scalable, physiologically relevant platform with promising applications in safety testing, disease modeling, and regenerative medicine.

**6) Figure legends**

1. Deleted sentence ‘Arrow: subculture duration.’ in Fig. 1.

2. Added sentence ‘(D) Comparison of epidermis thickness according to ALI culture duration (10 and 20 days).’ in Fig. 2.

3. Added sentence ‘Data are presented as mean values ± SEM. p value: *p < 0.05.’ in Fig. 2.

4. IF staining for (A) ΔNp63 and (B) KRT14 to identify hKERAs distribution in hiPSC-SKE. IF staining for (C) Vimentin, (D) PDGFRα, and (E) Fibronectin to identify hFIBROs in hiPSC-SKE. Scale bar: 100 µm. 🡪 IF staining for (A) KRT14 and (B) ΔNp63 identifying SB distribution in hiPSC-SKE according to ALI culture duration (10 and 20 days). IF staining for (C) KRT10 to identify SS distribution and (D) Loricrin to identify SC distribution. IF staining for (E) vimentin, (F) PDGFRα, and (G) fibronectin to identify hFIBROs in hiPSC-SKE at D30 (ALI culture for 20 days). Scale bar: 50 µm.

<Reviewers' comments>

Reviewer #1: All comments are being submitted in a separate file in order to respect the limit of characters.
Please find the attached document for answer the required questions. I don't have any concern about dual publication or research ethics.

The manuscript demonstrates a promising approach using hiPSC-derived cells to create a 3D skin model for irritation testing. However, there are significant areas that need improvement before it is ready for publication, primarily in the detail and clarity of the Materials and Methods and the Results sections, and in adding more quantitative data to support the conclusions. More concise writing and careful revision are necessary to enhance the clarity and improve the scientific rigor of the manuscript.

**Response to Reviewer:**

We sincerely thank the reviewer for taking the time and effort to critique our study. We tried our best to address each comment in an attentive manner by further clarifying our experimental results. We hope that the provided explanations and adaptations will further clarify our study and sufficiently address the reviewer’s concerns. Once again, we thank you for being a part of this review process.

**Minor**

**Clarity and conciseness:** In some sections, the writing could benefit from improved clarity and conciseness. Some sentences are quite long and complex, making it difficult to follow the line of reasoning.

**Example 1 (Abstract)**

Original: "Research on artificial skin has advanced significantly, supporting cosmetic ingredient evaluation and skin regeneration treatment development. Artificial skin-based tests are well established, with standardized guidelines and widespread use of three-dimensional (3D) skin equivalent models (SKE) constructed from human primary skin cells. However, in the case of primary cells, there are problems with supply due to limited patients and difficulties in genetic-specific research."

Revised: "Advances in artificial skin research support cosmetic ingredient evaluation and skin regeneration. While 3D skin equivalent models (SKES) from human primary cells are established, limitations exist due to donor availability and difficulties in genetic-specific studies."

**Example 2 (Introduction):**

Original: "Among the various applications of SKEs, skin irritation and corrosion testing are prominent (7-9). International guidelines for skin irritation testing are well-established, with the Organization for Economic Cooperation and Development test guideline 439 (OECD TG 439) serving as a key reference (7-9). This testing is critical for verifying the safety of products such as cosmetics, pharmaceuticals, and chemicals by evaluating whether a substance causes physical or chemical irritation or damage to the skin. And OECD TG 439 proposes a method that uses an artificial skin model to replace traditional animal testing."

Revised: "SKEs are widely used for skin irritation and corrosion testing, guided by international standards like OECD TG 439. This testing is crucial for evaluating the safety of cosmetics, pharmaceuticals, and chemicals, and OECD TG 439 promotes artificial skin models as a replacement for animal testing."

**Response 1:**

We apologize for the lack of clarity and readability in the original manuscript. In response to the reviewer’s valuable comments, we have thoroughly revised the manuscript to improve overall clarity and conciseness. Corrected sentences in the revised manuscript have been highlighted in blue color.

**Specific questions**

**Cell purity and characterization:** The authors claim high purity for the differentiated hFIBROs and hKERAs. What specific methods beyond FACS analysis were used to confirm the purity and absence of undifferentiated hiPSCs? Could you provide data on the percentage of cells expressing pluripotency markers in the final populations?

**Response 2:**

We appreciate reviewer comments that can improve the quality of the study. In response to the reviewer’s comment, we performed gel electrophoresis analysis for pluripotency markers to confirm the absence of undifferentiated PSCs in the differentiated hFIBROs and hKERAs. The results showed that pluripotency markers SOX2 and OCT4 were not expressed at the mRNA level in the differentiated cells. In addition, cell type–specific markers, which were not expressed in hiPSCs, were clearly expressed in the differentiated cells.

These findings have been included as Supplementary Figure 2, and the following sentence has been added to the Results section of the revised manuscript: “To further confirm the absence of residual undifferentiated cells in the differentiated hFIBROs and hKERAs, gel electrophoresis analysis for pluripotency markers was performed (Supple fig. 2). Pluripotent markers such as SOX2 and OCT4 were strongly expressed in undifferentiated hiPSCs but were undetectable in both hFIBROs and hKERAs. In contrast, fibroblast-specific markers (PDGFRα, Col3A1, and fibronectin) were exclusively expressed in hFIBROs (Supple fig. 2A), while keratinocyte-specific markers (KRT14 and ΔNp63) were detected only in hKERAs (Supple fig. 2B).”.

(Supplementary figure. 2)

**3D skin equivalent model (hiPSC-SKE) construction:** The protocol for hiPSC-SKE construction involves several steps. Could you explain in more detail the rationale behind the choice of specific media and growth factors at each stage? What optimization experiments were performed to determine the optimal cell densities and culture conditions for each layer?

**Response 3:**

We established the protocol for this study by referring to previously reported differentiation protocols for hiPSC-derived fibroblasts and keratinocytes (Kim et al., Stem cell research & therapy, 2018; Kim et al., Journal of Visulized Expriments. 2019; Domingues et al., Cells, 2022). Growth factors (EGF and BMP4) and insulin, known to promote differentiation into early skin cells, were applied to both hFIBRO and hKERA cultures. For induction toward hFIBRO, a higher concentration of FBS than that used for keratinocytes was continuously applied to promote differentiation into mesenchymal-like cells (Kim et al., Stem cell research & therapy, 2018). RA and BMP4, a key factor regulating keratinocyte proliferation and differentiation, was applied during hKERA culture (Metallo et al., Stem Cells, 2008). Additionally, CnT-07 medium was introduced to improve keratinocyte quality (Domingues et al., Cells, 2022). Compared to the previously used differentiation medium (KDM2 without RA) (Kim et al., Stem cell research & therapy, 2018), treatment with CnT-07 resulted in a more distinct keratinocyte-like morphology in hKERAs as well as enhanced proliferation.

(Comparioson of ‘KDM2 (w/o RA)’ and ‘CnT-07’ for differentiation of hKERA)

To construct the hiPSC-SKE, we optimized the differentiation protocol based on previously reports (Kajiwara et al., Stem Cell Reports, 2017; Domingues et al., Cells, 2022). Notably, unlike previous report (Domingues et al., Cells, 2022), we introduced an additional stabilization ((+) stabilization) step in which hKERAs were treated with CnT-Prime Airlift medium for 4 days prior to ALI culture. As a result, the hKERAs formed a uniform layer without clumping, leading to the successful establishment of a stable epidermal structure.

(Epidermis morphology according to stabilization of CnT-Prime Airlift media)

**Triton X-100 as a positive control:** Why was Triton X-100 chosen as the positive control for skin irritation? Are there other commonly used positive controls, and why was this one preferred? Were there any considerations regarding potential non-specific effects of Triton X-100 on cell viability?

**Response 4:**

According to the OECD TG 439 guideline for skin irritation testing, sodium dodecyl sulfate (SDS) or Triton X-100, both known to induce cytotoxicity, are used as positive controls. After treatment with the positive control, tissue damage in the skin equivalents (SKEs) is assessed using an absorbance-based cell viability assay. Triton X-100 is a nonionic detergent that specifically binds to lipids, disrupts cell membranes, and induces cell death. It is widely used as a positive control in skin irritation tests because it induces reproducible skin irritation under standardized conditions with minimal nonspecific cytotoxicity. Reflecting the reviewer's comment, the related information has been included in the Results section of the revised manuscript.

**Statistical analysis:** The statistical analysis uses one-way ANOVA. Are there post-hoc tests used to determine which groups are significantly different from each other? A more detailed explanation of the statistical methods is warranted.

**Response 5:**

Reflecting the reviewer’s comment, we have revised the manuscript to include the following sentence “Data were presented as mean ± SEM, and the statistical significance of the experimental results was calculated using either a T-test or one-way ANOVA with Tukey’s method for comparison between groups.”.

**Mechanism of Triton X-100-induced damage:** The study shows that Triton X-100 causes damage to the epidermis. However, what are the mechanisms underlying this damage? Additional investigation into cell death pathways and signaling is needed.

**Response 6:**

Thank you for your valuable comment. As correctly noted, Triton X-100 is well known to induce cell damage primarily through its interaction with lipid components of the plasma membrane (Mattei et al., Chem Phys Lipids., 2017; Rusanov et al., Bull Exp Biol Med., 2017; Ip et al., Skin Res Technol., 2024). In our revised manuscript (‘Result’ section), we have included an explanation of Triton X-100 known mechanism of action, as follows:

“Triton X-100 is a nonionic detergent that disrupts cell membranes by specifically binding to membrane lipids, damaging the structural integrity of the membrane. This increases membrane permeability and causes ion imbalance, ultimately disrupting cellular homeostasis and inducing cell death. Additionally, increased cellular stress damages the mitochondrial membrane potential, further promoting cell death (33-36). Triton X-100 induces reproducible skin irritation under standardized conditions with minimal nonspecific cytotoxicity, making it a widely used positive control in skin irritation testing.”.

Since the main objective of our study was to demonstrate the applicability of hiPSC-SKE for skin irritation testing, we followed established guidelines by conducting cell viability assays and histological analyses (Liao et al., Toxicol In Vitro., 2021; Jung et al., Toxicol In Vitro., 2014). Our results showed that Triton X-100 penetrated the outermost epidermal layer of the hiPSC-SKE, leading to significant cell death and reduced O.D values in the viability assay (Figs. 4C-4E).

**Methods section:** The methods section could benefit from more detailed information regarding the specific concentrations of reagents used, the duration of each treatment step, and the criteria used for assessing the success of the differentiation protocols.

**Response 7:**

Thanks for the detailed advice from the reviewer. The original manuscript lacked clarity, which may have caused some difficulty in understanding. In the revised manuscript, we have provided detailed information on specific concentrations, durations, and criteria.

**hiPSC generation:** The protocol for generating hiPSCs from human dermal fibroblasts is briefly described. Specify the episomal vectors used, the duration of reprogramming, and the selection methods used to enrich for hiPSC colonies.

**Response 8:**

In response to the reviewer’s comments, we added the following sentence to the Materials and Methods section of the revised manuscript: “(Generation of human induced pluripotent stem cells (hiPSCs)) 1.5 × 10⁶ human dermal fibroblasts (BJ cells; ATCC, VA, USA) were transfected with episomal vectors (pCLXE-hOCT4/p53, pCLXE-hSOX2/KLF4, and pCLXE-hL-MYC/LIN28A; Addgene, MA, USA) using the P2 Primary Cell 4D-Nucleofector Kit (Lonza, Basel, Switzerland) according to the manufacturer’s protocol (22, 23). The mixture of cells and vectors was transferred into a Nucleocuvette Vessel and subjected to electroporation. Transfected cells were plated onto 1:100 Matrigel (Corning, NY, USA)-coated 6-well plates and cultured for 2 days in Dulbecco’s Modified Eagle’s Medium (DMEM; Gibco, NY, USA) supplemented with 10% fetal bovine serum (FBS; Sigma-Aldrich, St. Louis, MO, USA), 0.1 mM non-essential amino acids (NEAA; Gibco), and 1% penicillin/streptomycin (P/S; Gibco). After 2 days, the medium was replaced with TeSR-E7 medium (STEMCELL Technologies, Vancouver, Canada), which was changed daily. Cells were cultured for an additional 10–14 days until PSC-like colonies appeared. After picking the PSC-like colonies, they were seeded onto Matrigel-coated 4-well plates in mTeSR1 (STEMCELL Technologies Inc; Vancouver, BC, Canada) medium supplemented with 10 µM Y-27632 (Tocris Bioscience, Bristol, UK). The medium was changed daily with fresh mTeSR1 during subsequent culture.”.

**Differentiation protocols:** The protocols for differentiating hiPSCs into hFIBROs and hKERAs are incomplete. Specify the exact composition and concentrations of all growth factors and supplements in the various media (FDM1, FDM2, FDM3, KDM1, KDM2). Provide details on the cell culture conditions (temperature, CO2 concentration, etc.).

**Response 9:**

Thank you for the detailed advice from the reviewer. We have detailed the differentiation protocols in the Materials and Methods section of the revised manuscript and indicated the media used at each differentiation stage (FDM1, FDM2, FDM3, KDM1, KDM2) in the protocol image of Figure 1.

Figure. 1 in revised version

**hiPSC-SKE construction:** Describe the process of creating the hiPSC-SKE model in more detail. What type and concentration of collagen was used? What was the seeding density of the hKERAs? Were any additional factors used to promote keratinization?

**Response 10:**

In response to the reviewer’s comment, we have revised the manuscript to provide a more detailed description of the hiPSC-SKE fabrication process. The specific information of type I collagen (Corning, NY, USA, Cat# 354249) used for dermal layer fomation have been clarified. We have specified detailed information on the collagen type I (Corning, NY, USA, Cat# 354249) used in our revised manuscript. For dermis layer construction, collagen was used at a final concentration of 4.5 mg/ml. Additionally, 1.5 × 10⁶ hKERAs were seeded onto the dermal layer to reconstruct the epidermis. To enhance keratinization, we used CnT-Prime Airlift Medium containing 1 mM calcium chloride (Domingues et al., Cells, 2022). Additionally, a stabilization period ((+) stabilization) was included to improve epidermal layer integrity before initiating the air-liquid interface culture.

(Epidermis morphology according to stabilization of CnT-Prime Airlift media)

**Immunofluorescence:** Clarify the antibodies used (company, catalog numbers). What was the counterstain used?

**Response 11:**

Reflecting the reviewer’s comment, we have added detailed information on the antibodies used, including the manufacturer, catalog numbers, and dilution ratios: “As primary antibodies, anti-Vimentin (abcam, Cat# ab8978; 1:200), anti-Fibronectin (abcam, Cat# ab2413; 1:200), anti-ΔNp63 (abcam, Cat#203826; 1:100), anti-KRT14 (abcam, Cat# ab181595; 1:200), anti-platelet-derived growth factor receptor α (PDGFRα; abcam, ab32570), anti-KRT10 (abcam, Cat# ab76318; 1:200), and anti-Loricirin (abcam, Cat# ab198994; 1:100) were used.”. We have also specified that DAPI (4′,6-diamidino-2-phenylindole; Sigma-Aldrich, Cat# D9542) was used as the nuclear counterstain. Additionally, we noted that the fluorescent dye colors in the images were adjusted for visual clarity.

**Results**

The results are presented in a descriptive manner; some sections may be improved with more concise writing and more direct presentation of the findings.

The results section needs more quantitative data. Present specific percentages of cells positive for the different markers (vimentin, fibronectin, KRT14, ANp63) in the FACS and immunofluorescence analyses. Quantify the "significant morphological changes" observed in the hiPSC-SKE. Include numerical data on the thickness of the epidermal layers at different time points.

The figure legends provide some descriptive information, but more quantitative data should be incorporated where applicable. For example, specific measurements of epidermal thickness in Figure 2 should be included. The legends for Figures 3 and 4 would be improved by specifying the antibodies used and the types of staining techniques.

**Response 12:**

We sincerely thank the reviewer for the valuable comment. To address this, we analyzed the distribution of Vimentin⁺ and ΔNp63⁺ cells based on immunofluorescence (IF) staining to quantify the purity of hFIBROs and hKERAs, respectively. The results demonstrated that both cell types exhibited over 90% marker-positive populations, consistent with the FACS analysis. These quantitative data have been added as Supplementary Figure 1, and the following sentence has been included in the revised ‘Results’ section: “The purity of hFIBROs was assessed by analyzing the density of vimentin+ cells per field, which was 97.01 ± 0.73% (Supple fig. 1A).” and “The purity of hKERAs was assessed by analyzing the density of ΔNp63+ cells per field, which was 98.09 ± 0.24% (Supple fig. 1B).”.

Supplementary figure 1 in revised version

Furthermore, we quantified the thickness of the epidermal layer at different ALI culture time points, showing a statistically significant increase in the ALI-20 days compared to ALI-10 days (ALI-10 days: 38.82 ± 2.20 μm vs. ALI-20 days: 63.62 ± 8.06 μm). These quantitative data have been added as Fig. 2D, and the following sentence has been included in the revised ‘Results’ section: “Keratinization of the epidermis was more prominent at ALI-20 days than at ALI-10 days of ALI culture. The epidermal thickness was significantly increased at ALI-20 days (ALI-10 days: 38.82 ± 2.20 μm vs. ALI-20 days: 63.62 ± 8.06 μm), accompanied by thickening of the stratum basale (SB) and stratum spinosum (SS)-like layers.”.

Figs. 2C and 2D in revised version

To improve the quality of the hiPSC-SKE, additional experiments were performed, and the information of revised results were more clearly described in the legend of Fig. 3.

Fig.3 in revised version

**Discussion:** The discussion section could be strengthened by including a more detailed comparison to other relevant research and a more thorough discussion of the potential limitations of the study. **The clinical implications of the findings should also be more explicitly stated.**

Comparison to existing SKEs: How do the characteristics of the hiPSC-SKE compare to commercially available SKEs (e.g., EpiDerm, Episkin) in terms of morphology, marker expression, and response to irritation testing?

Limitations and future directions: The authors mention limitations of using primary cells. What are the limitations of the hiPSC-SKE model presented in this study? What are the next steps or future directions of this research to overcome these limitations?

**Response 13:**

Reflecting the reviewer’s comment, we have thoroughly revised the ‘Discussion’ section. In particular, we included a comparison between hiPSC-SKE models and existing SKE models, focusing on the immaturity of iPSC derived cells, a known limitation. Additionally, we elaborated on future directions to enhance tissue maturation and address batch to batch variability. The relevant content has been added to the ‘Discussion’ section of the revised manuscript as the sentence ‘hiPSC-SKEs demonstrated marker expression and irritation responses comparable to primary skin cell-based SKEs, supporting their potential as reliable alternatives. hiPSCs offer advantages such as overcoming limitations in cell supply, cost, and donor variability. However, a key challenge remains as hiPSC-derived cells often exhibit an immature phenotype (42, 43). Similarly, hiPSC-derived skin organoids lack structural completeness, such as Collagen VII and immune or endothelial cells (44, 45). This immaturity may also affect hiPSC-derived skin cells, potentially explaining the need for ALI culture durations exceeding 20 days. Moreover, SKEs generated from primary skin cells often display a thicker SC compared to hiPSC-SKEs (20, 24). To enhance model maturity, future studies should incorporate additional skin constituent cells to better reflect in vivo conditions. Batch-to-batch variability during hiPSC differentiation also poses challenges for reproducibility. To address this, we are exploring previously reported droplet-based bioprinting and nebulization technologies to standardize hiPSC-SKE fabrication. In summary, the hiPSC-SKE model offers a scalable, physiologically relevant platform with promising applications in safety testing, disease modeling, and regenerative medicine.’.

Reviewer #2: In the manuscript by Shin and colleagues human induced pluripotent stem cells were produced and were differentiated into fibroblasts and keratinocytes. Those cells were then used to establish 3D skin equivalents. Establishing high trough put in vitro skin models is important to meet the high demand of new models needed to replace and reduce animal experiments, especially with regard to chemical testing.
The manuscript is nicely written follows a clear path presenting results, although there are some major draw backs

**Response to Reviewer:**

We sincerely thank the reviewer for taking the time and effort to critique our study. We tried our best to address each comment in an attentive manner by further clarifying our experimental results. We hope that the provided explanations and adaptations will further clarify our study and sufficiently address the reviewer’s concerns. Once again, we thank you for being a part of this review process.

1. In General, the writing is not clear, especially in the methods section. The English should be edited by a native speaker.

**Response 1:**

We apologize for the lack of clarity and readability in the original manuscript. In response to the reviewer’s valuable comments, we have thoroughly revised the manuscript to improve overall clarity and conciseness. Corrected sentences in the revised manuscript have been highlighted in blue color.

2. Methods: Generation of hi-PSC should be explained in more detail.

**Response 2:**

In response to the reviewer’s comments, we added the following sentence to the Materials and Methods section of the revised manuscript: “(Generation of human induced pluripotent stem cells (hiPSCs)) 1.5 × 10⁶ human dermal fibroblasts (BJ cells; ATCC, VA, USA) were transfected with episomal vectors (pCLXE-hOCT4/p53, pCLXE-hSOX2/KLF4, and pCLXE-hL-MYC/LIN28A; Addgene, MA, USA) using the P2 Primary Cell 4D-Nucleofector Kit (Lonza, Basel, Switzerland) according to the manufacturer’s protocol (22, 23). The mixture of cells and vectors was transferred into a Nucleocuvette Vessel and subjected to electroporation. Transfected cells were plated onto 1:100 Matrigel (Corning, NY, USA)-coated 6-well plates and cultured for 2 days in Dulbecco’s Modified Eagle’s Medium (DMEM; Gibco, NY, USA) supplemented with 10% fetal bovine serum (FBS; Sigma-Aldrich, St. Louis, MO, USA), 0.1 mM non-essential amino acids (NEAA; Gibco), and 1% penicillin/streptomycin (P/S; Gibco). After 2 days, the medium was replaced with TeSR-E7 medium (STEMCELL Technologies, Vancouver, Canada), which was changed daily. Cells were cultured for an additional 10–14 days until PSC-like colonies appeared. After picking the PSC-like colonies, they were seeded onto Matrigel-coated 4-well plates in mTeSR1 (STEMCELL Technologies Inc; Vancouver, BC, Canada) medium supplemented with 10 µM Y-27632 (Tocris Bioscience, Bristol, UK). The medium was changed daily with fresh mTeSR1 during subsequent culture.”.

3. Formation of 3D SKE: Although there is a good separation into dermis and epidermis, the epidermis is not differentiated properly. There is no stratum granulosum and no stratum corneum formed. Differences in stratum basale and spinosum in H&E staining are only minor. Skin models should be improved so that sublayers of the epidermis form properly as seen in 3D SKE made from primary skin cells. Have different timings been tested? 30 days are quite long compared to other published 3D skin models.

**Response 3:**

We sincerely thank the reviewer for the valuable comment. We agree with the concern and apologize for the potential confusion caused by the low image quality of the epidermal layer. To solve the issue, we performed additional experiments using newly differentiated hFIBROs and hKERAs to reconstruct the hiPSC-SKE. As described in the manuscript, we maintained the same culture duration and cell numbers based on our established protocol, which was developed with reference to previously published studies.

As a result, the epidermis at ALI-20 days (D30) showed more distinct morphology and a significantly increased thickness compared to ALI-10 days (D20). Notably, a stratum corneum (SC)-like structure was observed at the air-exposed surface on ALI-20 days. To further investigate the degree of differentiation, we performed additional immunofluorescence staining for representative markers of each epidermal layer. KRT14 and ΔNp63, known markers for keratinocytes in the basal layer (SB), were strongly expressed in the lower part of the epidermis. Especially, ΔNp63 expression gradually decreased toward the upper layers at ALI-20 days compared to ALI-10 days. KRT10, a marker for the spinous layer (SS), was strongly detected above the SB, and the expression density was thicker at ALI-20 days than at ALI-10 days. Loricrin, a late differentiation marker expressed in the stratum corneum (SC), was not detected at ALI-10 days, but showed a clear, band-like expression pattern at ALI-20 days. These results confirm that the hiPSC-SKE recapitulates the epidermal differentiation process, similar to that observed in 3D SKEs generated from primary skin cells.

Figs. 2C and 2D in revised version

Fig.3 in revised version

As the reviewer pointed out, the keratinization period in our model is somewhat longer compared to previously reported SKE models based on primary skin cells. We consider this to reflect the immaturity of hiPSC-derived cells compared to *in vivo* adult cells (Koivumäki et al., Frontiers in physiology, 2018; Tao et al., Cell Stem Cell, 2016; Sandoval et al., The Journal of investigative dermatology, 2023; Oceguera et al., Frontiers in cell and developmental biology, 2022). This immaturity can also affect hiPSC-derived skin cells and may explain why other hiPSC-SKE studies require ALI culture periods exceeding 20 days (Kajiwara et al., Stem Cell Reports, 2017; Domingues et al., Cells, 2022). Furthermore, SKEs generated from primary skin cells often have a thicker stratum corneum compared to those derived from hiPSCs. These points have been addressed in the ‘Discussion’ section.

4. Further, proper epidermal differentiation should not only be assessed by H&H staining but also IHC, staining for markers specific for certain sublayers, e.g. Fillagrin, Involucrin, Ki-67 or different keratins.

**Response 4:**

Reflecting the reviewer’s comment, we performed additional IF staining for representative markers (KRT14, ΔNp63, KRT10, and Loricrin) of each epidermal layer. The results are consistent with those described in ‘Response 3,’ and the related data are presented in the revised version of Figs. 3A–3D.

Fig.3 in revised version
